# Three-dimensional analysis of hard and soft tissue changes in skeletal class II patients with high mandibular plane angle undergoing surgery

**DOI:** 10.1038/s41598-024-51322-1

**Published:** 2024-01-30

**Authors:** Caixia Zhang, Tong Lu, Lichan Wang, Juan Wen, Ziwei Huang, Shuang Lin, Yiwen Zhou, Guifeng Li, Huang Li

**Affiliations:** 1 Nanjing Stomatological Hospital, Affiliated Hospital of Medical School, Research Institute of Stomatology, Nanjing University, Nanjing, China; 2 Nanjing Lishui Stomatological Hospital, Nanjing, China

**Keywords:** Oral anatomy, Oral diseases

## Abstract

This study aimed to study 3-dimensional (3D) changes of hard and soft tissues of skeletal class II patients after 2-jaw surgery and genioplasty. 32 adult patients diagnosed with mandibular hypoplasia who underwent 2-jaw surgery of maxillary impaction, mandibular advancement and genioplasty were enrolled. Cone-beam computed tomography and 3D stereophotogrammetry was conducted 1 week before and 6 months after surgery. Dolphin imaging software was used to establish a 3D digitizing model and 3D measurement system. Paired t-test was performed to compare the values before and after surgery. Pearson’s correlation test assessed the degree of correlations between hard and soft tissue change. The mean impaction of the maxilla was 2.600 ± 3.088 mm at A. The mean advancement of the mandible was 7.806 ± 2.647 mm at B. There was a significant upward and forward movement for most landmarks of the nose and lip, while a significant decrease in nasal tip height (lateral view), upper lip height, and upper and lower vermilion height. The nose's width was significantly increased. For maxillary, Sn, Ac-r, Ac-l, and Ls demonstrated a significant correlation with A and U1 in the anteroposterior axis. However, there were no significant correlations among them in the vertical axis. For mandibular, Li demonstrated a significant correlation with L1 in the anteroposterior axis specifically for the mandible. Notably, correlations between the landmarks of the chin's hard and soft tissues were observed across all axes. The utilization of 3-D analysis facilitated a quantitative comprehension of both hard and soft tissues, thereby furnishing valuable insights for the strategic formulation of orthognathic treatment plans targeting patients with skeletal class II conditions.

## Introduction

Facial appearance has become increasingly important in our modern society and is crucial in social interactions^1^. Nowadays, more and more adults are seeking medical advice for improving dental and facial aesthetics. Notably, Angle Class II malocclusions adult patients with skeletal dysmorphology have been performed with combined orthodontic and orthognathic surgical treatment for years. Treatment planning for these patients should correct the malocclusion involving the stomatognathic function and improve facial esthetic^2,3^. Since the patient’ postoperative satisfaction depends on the soft tissues’ position to a great extent, it is important for the clinician to predict soft tissue changes when treatment planning.

Many authors have studied the soft-to-hard tissue changes after mandibular advancement surgery. However, most previous studies used 2D lateral cephalograms, and skeletal changes following orthognathic surgery were evaluated only in an anteroposterior or vertical dimension, which were not assessed in a transverse dimension from a frontal aspect^4,5^. Only a few studies of 3D soft tissue changes used CBCT and facial scan imaging after orthognathic surgery in mandibular retrognathia^6,7^, but they seldom studied the relationships after two jaw surgeries. These investigations found a correlation between mandibular advancement and volumetric changes in the hard tissues, but there was not a specific ratio between the soft and hard tissues. It is time to optimize these techniques for this kind of elective procedures lagged behind during COVID-19 pandemic^8,9^. Therefore, it is meaningful to study the changes and ratios of soft-to-hard tissue from three dimensions for the patients treated with a LeFort I osteotomy, a bilateral sagittal split osteotomy (BSSO), and a genioplasty.

Cone-beam computed tomography (CBCT) provides three-dimensional (3D) information about deep skeletal structures and superficial skin but has disadvantages in soft tissue evaluation due to a low resolution with large slice gaps and deformations of the soft tissues of the chin due to the device’s chin holder^10,11^. Additionally, by using 3D stereophotogrammetry, texture and color information of the face can be readily obtained in high resolution without additional radiation hazards, along with advantages such as a short scan time, no risk to the naked eye, and easy operability^12^. Therefore, CBCT and 3D stereophotogrammetry have been combined to evaluate the relationship between hard and soft tissue changes^13,14^. However, most 3-D studies of hard and soft tissue changes after orthognathic surgery focused on skeletal class III patients^13,14^. A few researchers have focused on skeletal class II patients, but only mandibular advancement was performed^6,15^.

In this study, we aimed to study the 3D changes and correlation between hard and soft tissue movements in patients with skeletal Class II malocclusion with a severe retruded mandible and a vertical growth pattern which had received LeFort I osteotomy for maxillary impaction, a BSSO for mandibular advancement and rotation, and a genioplasty for correcting the skeletal deformities using CBCT and 3D stereophotogrammetry.

## Methods

### Subjects

Skeletal Class II patients who had received LeFort I osteotomy, BSSO, and genioplasty only by the same surgeon between 2017 and 2022 were screened. Similar surgical techniques were used in all cases, and rigid internal fixation was applied, according to Tulasne^16^. The following criteria were considered for this study: (1) Patients must have complete pre- and postoperative stereophotogrammetry records (postoperative records must be at least 6 months after surgery after debonding). (2) There was no presence of a congenital anomaly or craniofacial deformity, no history of accidents or trauma, and no presence of severe asymmetry at Me (< 5 mm to the midsagittal plane). (3) Maxillary impaction surgery with one piece Le Fort I osteotomy and mandibular advancement bilateral sagittal split ramus osteotomy with genioplasty was performed on all patients during a correction of skeletal class II malocclusion. (4) Patients underwent surgery at the same center (Nanjing Stomatological Hospital, Medical School of Nanjing University, Department of Oral and Maxillofacial Surgery). (5) Similar rigid fixation methods were performed for stabilization after the surgery. Exclusion criteria were as follows: (1) history of accidents or trauma, (2) presence of severe asymmetry at Me (> 5 mm to the midsagittal plane), (2) subjects with a body mass index greater than 30 kg/m^2^, and (3) subjects with increased or decreased body weight more than 5 kg before and after surgery. Thus, 32 adult Chinese subjects (15 men and 17 women; mean age, 21.73 ± 3.87 years at the start of treatment) (the power calculated by PASS 15.0 software was more than 90%) were included in this study. This retrospective study was registered and approved by the Ethics Committee of Nanjing Stomatological Hospital. Furthermore, all methods were performed following the approved guidelines and regulations, all subjects had signed the informed consent form for both study participation and publication of identifying information/images**.**

### Data acquisition

CBCT and 3D stereophotogrammetry were taken 1 week before and 6 months after surgery (without braces debonding) (T0 and T1, respectively). The CBCT (KaVo 3D eXam, USA) image data was obtained within 1 week before surgery. The CBCT imaging conditions were as follows: voxel size: 0. 25 mm; field of view: 16 × 13 cm; tube voltage: 120 kVp, and tube current: 5.0 mA. Digital Imaging and Communications in Medicine (DICOM) data from multislice CT images were reconstructed and analyzed using image-processing software Dolphin (version 11.8; Dolphin Imaging and Management Solutions, Chatsworth, CA, USA). Additionally, 3D stereophotogrammetry images were obtained using the 3dMDface system (3dMDface LLC, Atlanta, GA, USA), with a natural head position, relaxed facial expression, and eyes looking straight ahead^17^. The data of 3dMD was saved in the BMP format. Both formats were transformed into Dolphin software and integrated into a complete 3D digital image before measuring. Five landmark points (bilateral outer canthus, the tip of the nose, and bilateral angulus oris) in the soft face were selected to manually match the 3dMD images and CBCT scans. The final images were presented for measurement.

### Landmarks, planes, and measurements

**Figure 1:**
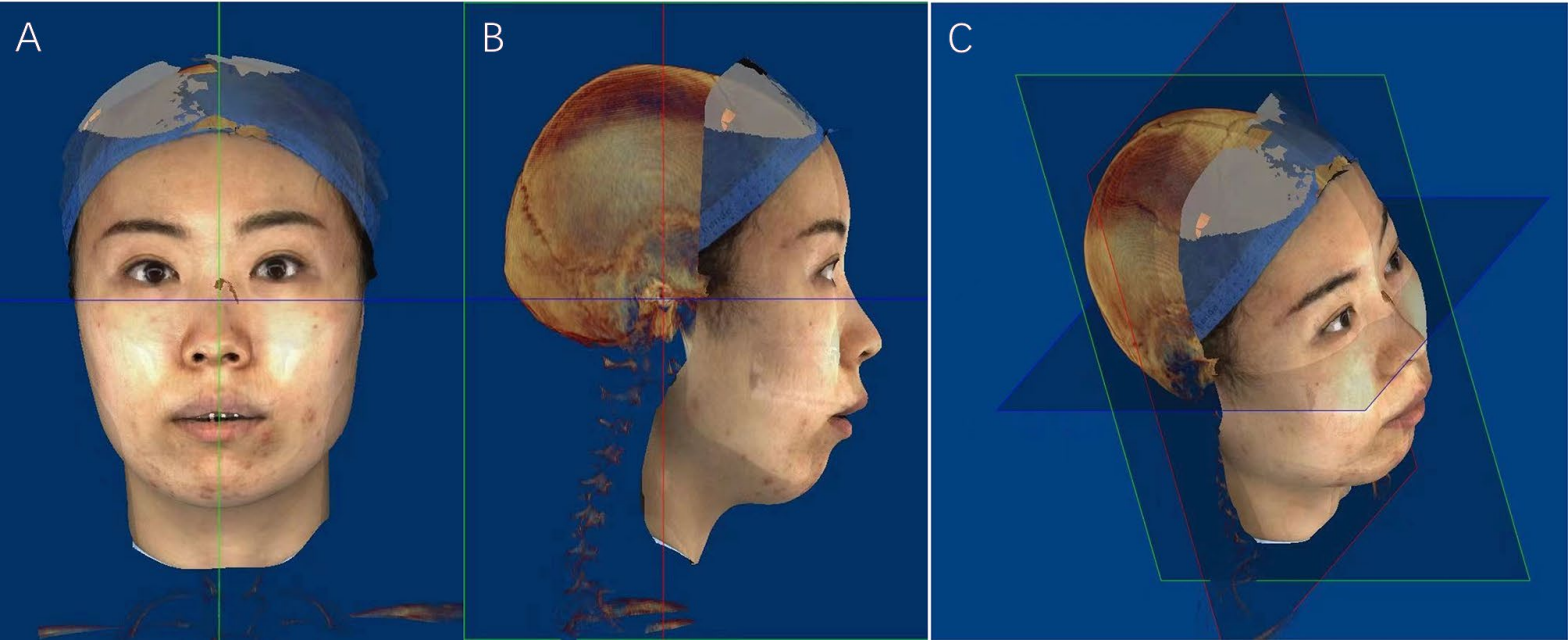
Superimposed 3D image of CBCT and facial scan and 3-dimensional coordinate system.

A 3D coordinate system was established in CBCT images as follows: the X-direction represents the horizontal plane(x), passing through the left and right orbitale (the lowest point on the inferior margin of the orbit) and the right porion (the midpoint of the upper contour of the external auditory canal); the Y-direction which represents the anteroposterior plane(y) is vertical to the X-direction, passing through the right porion; and the Z-direction is the vertical direction passing through the nasion (Fig. 1).

**Figure 2:**
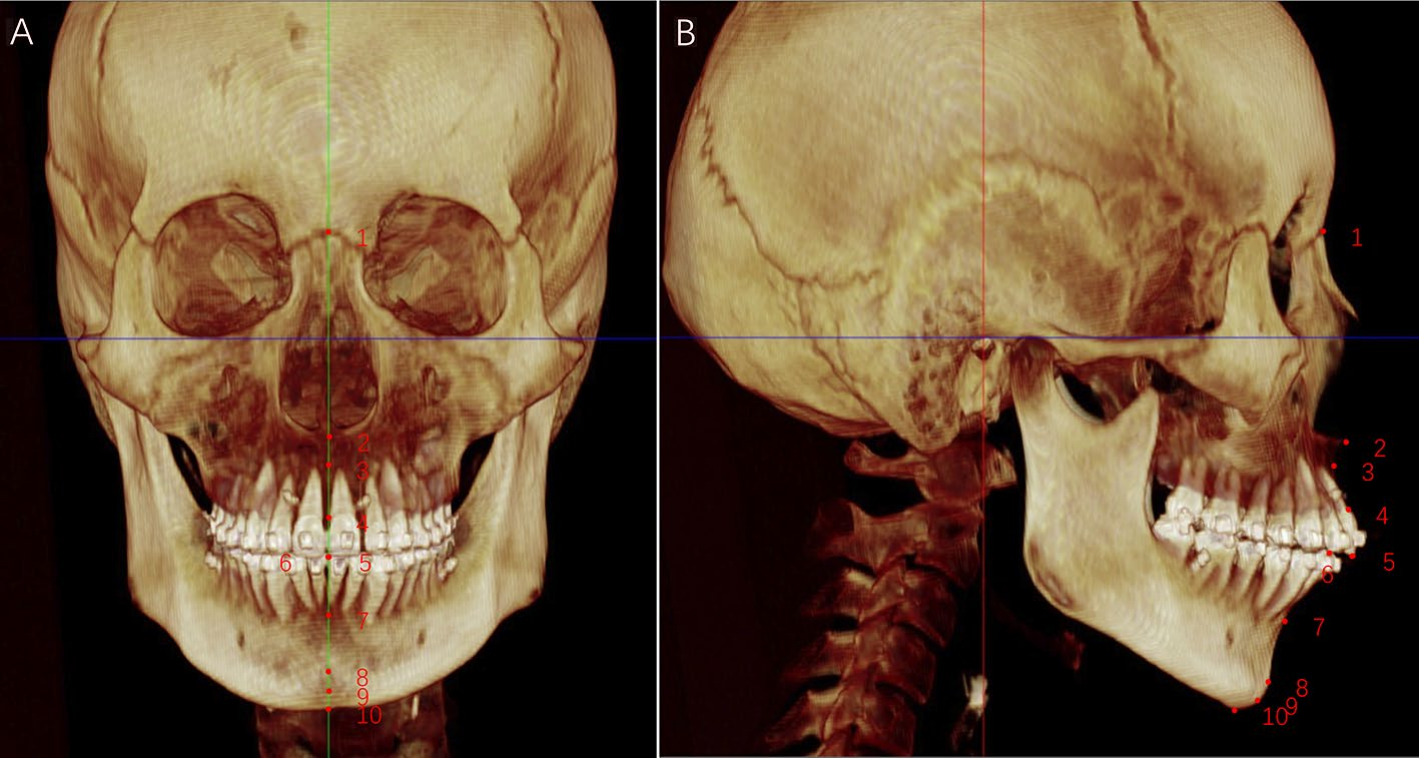
Skeletal landmarks on CBCT images: 1, Nasion (N); 2, Anterior nasal spine (ANS); 3, A Point (**A**); 4, U1 Labial Gingival Border (U1L); 5, U1 Tip (U1); 6, L1 Tip (L1); 7, B point (**B**); 8, pogonion (Pog); 9, Anatomical Gnathion (Gn); 10, menton (Me). See also Table 1 for the description of 3D CBCT landmarks.

**Figure 3:**
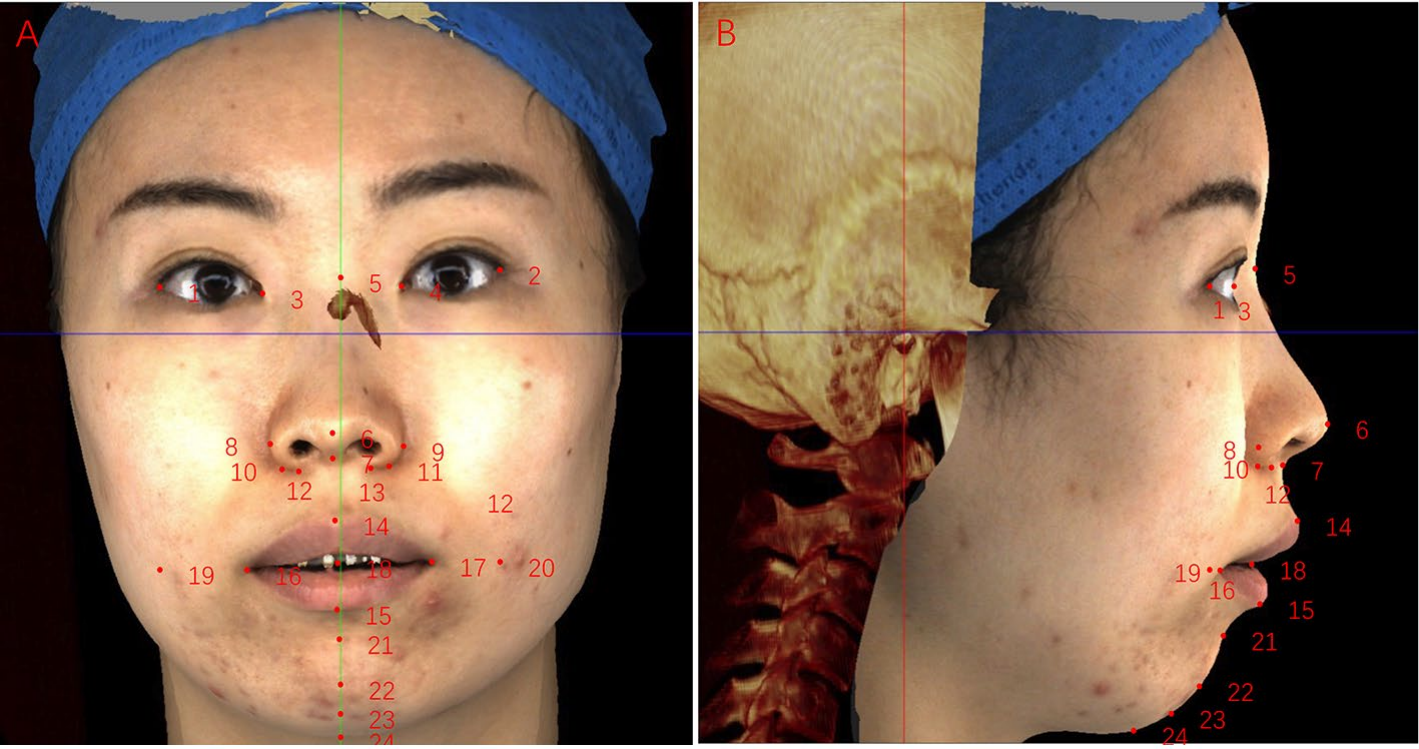
Soft tissue landmarks on facial scan images. 1, Exocanthion-right(Ex-r); 2, Exocanthion-left(Ex-l); 3, Endocanthion-right (En-r); 4, Endocanthion-left (En-l); 5, Nasion′ (N′); 6, Pronasale (Prn); 7, Subnasale(Sn); 8, Alare-right(Al-r); 9, Alare-left (Al-l); 10, Alare curvature-right (Ac-r); 11, Alare curvature-left (Ac-l); 12, Subalare-right (Sbal-r); 13, Subalare-left (Sbal-l); 14, Labrale superior (Ls); 15, Labrale inferior (Li); 16, Cheilion-right (Ch-r); 17, Cheilion-left (Ch-l); 18, Stomion (Sto); 19, Cheek point-right (Ck-r); 20, Cheek point-left (Ck-l); 21, B′ point (B′’); 22, Pogonion′ (Pog′), 23, Gnathion′ (Gn′); 24, Menton′ (Me′). See also Table 2 for the description of 3D soft tissue landmarks.

A total of 10 skeletal and 24 soft tissue landmarks were identifiedand measured in the 3D coordinate values before and after surgery (Figs. 2 and 3, Tables 1 and 2). A positive ( +) sign indicated the point deviated to the left, anterior, and upper side to N of the subject. A negative ( −) sign indicated the opposite. The changes in the landmarks, correlation coefficients (p), and soft-to-hard tissue movement ratios were evaluated in x, y and z axis. Furthermore, 14 linear measurements of soft tissue were assessed (Table 3).

**Table 1:** Definitions of hard tissue landmarks.

	Abb.	Definition
Nasion	N	The anterior point of the intersection between the nasal and frontal bones
Anterior nasal spine	ANS	The tip of the anterior nasal spine (sometimes modified as the point on the upper or lower contour of the spine where it is 3 mm thick; see Harvold analysis)
A Point	A	The innermost point on the contour of the premaxilla between anterior nasal spine and the incisor tooth
U1 Labial Gingival Border	U1L	Labial cemento-enamel junction of the upper central incisor
U1 Tip	U1	Incisor tip of the upper central incisor
L1 Tip	L1	Tip of the lower central incisor
B Point	B	The innermost point on the contour of the mandible between the incisor tooth and the bony chin
Pogonion	Pog	The most anterior point on the contour of the chin
Anatomical Gnathion	Gn	The midpoint between the most anterior and inferior point on the bony chin
Menton	Me	The most inferior point on the mandibular symphysis (i.e., the bottom of the chin)

**Table 2:** Definitions of soft tissue landmarks.

	Abb.	Definition
Exocanthion-right	Ex-r	The soft tissue point located at the outer commissure of the right eye fissure
Exocanthion-left	Ex-l	The soft tissue point located at the outer commissure of the left eye fissure
Endocanthion-right	En-r	The soft tissue point located at the inner commissure of the right eye fissure
Endocanthion-left	En-l	The soft tissue point located at the inner commissure of the left eye fissure
Nasion′	N′	The point in the midline of both the nasal root and the frontonasal suture
Pronasale	Prn	The most prominent point on the nasal tip
Subnasale	Sn	The midpoint on the nasolabial soft tissue contour between the columella crest and the upper lip
Alare-right	Al-r	The most lateral point on the right alar contour
Alare-left	Al-l	The most lateral point on the left alar contour
Alare curvature-right	Ac-r	The point located at the facial insertion of the right alar base
Alare curvature-left	Ac-l	The point located at the facial insertion of the left alar base
Subalare-right	Sbal-r	The point of the end of the alare extends to the facial skin
Subalare-left	Sbal-l	The point of the end of the alare extends to the facial skin
Labrale superior	Ls	The midpoint of the vermilion line of the upper lip
Labrale inferior	Li	The midpoint of the vermilion line of the lower lip
Cheilion-right	Ch-r	The point located at the right labial commissure
Cheilion-left	Ch-l	The point located at the left labial commissure
Stomion	Sto	The midpoint between the lower border of the upper lip and upper border of the lower lip
Cheek point-right	Ck-r	The point where a vertical line from exocanthion and a horizontal line from cheilion meet on the right side
Cheek point-left	Ck-l	The point where a vertical line from exocanthion and a horizontal line from cheilion meet on the left side
B′ point	B′	The most deepest point from lateral view, on the facial midline, between the lower lip and chin
Pogonion′	Pog′	The most anterior midpoint of the chin
Gnathion′	Gn′	The midpoint between the most anterior and inferior point of the soft tissue chin
Menton′	Me′	The lowest point on the midline of the chin

**Table 3:** Description of soft tissue linear measurements in the study.

Landmarks	Definition
Width of Exocanthion (Ex-r to Ex-l)	Distance between Exocanthion of the deviated side and contralateral side
Width of Endocanthion (En-r to En-l)	Distance between Endocanthion of the deviated side and contralateral side
Height of the nose(N′–Sn)	Distance between Nasion′ and Subnasale
The length of the bridge of the nose(N-Prn)	Distance between Nasion′ and Pronasale
height of nasal tip(front view)	Distance between Subnasale and Pronasale on the Z-direction
height of nasal tip(lateral view)	Distance between Subnasale and Pronasale on the Y-direction
Width of Alare (Al-r to Al-l)	Distance between nasal Alare of the deviated side and contralateral side
Width of nasal Subalare (Sbal-r to Sbal-l)	Distance between nasal Subalare of the deviated side and contralateral side
Width of alar curvature (Ac-r to Ac-l)	Distance between nasal alare curvature of the deviated side and contralateral side
Upper lip height (Sn–Stms)	Distance between subnasale and stomion superius
Lip width (Ch dev–Ch ctl)	Distance between cheilion of the deviated side and contralateral side
Upper vermilion height (Ls–Stms),	Distance between labrale superius and stomion superius
Lower vermilion height (Li–stmi)	Distance between labrale inferius and stomion inferius

The datasets used during the current study available from the corresponding author on reasonable request.

### Statistical analysis

All measurements were conducted by one investigator and repeated after 2 weeks, and there was no difference between the two assessments revealed by a paired t-test (*p* > 0.05). The second set of measurements was used for the following statistical analysis. A paired t-test was conducted to compare the values between T1 and T2. And a Pearson’s correlation test was performed to assess the degree of correlations between hard and soft tissue changes after orthognathic surgery. Also, soft-to-hard tissue movement ratios were defined. *p* < 0.05 was defined as statistically significant.

## Results

### Changes in hard tissue landmarks

In the transverse axis, since no severe asymmetry was presented at Me in our patients, there were no significant changes in all hard tissue landmarks; (Table 4).

**Table 4:** Changes in hard tissue landmarks on CBCT images.

Landmarks	ΔT2-T1(transverse)			ΔT2-T1(anteroposterior)			ΔT2-T1(vertical)		
	Mean	SD	*p* value	Mean	SD	*p* value	Mean	SD	*p* value
N	0.000	0.000	−	0.200	1.155	0.335	− 0.231	0.710	0.075
ANS	0.000	0.000	−	− 0.991	1.757	0.003	1.269	1.975	0.001
A	− 0.025	0.701	0.166	− 0.675	1.810	0.043	2.600	3.088	0.000
U1L	− 0.006	0.702	0.131	0.247	1.995	0.489	3.806	2.491	0.000
U1	− 0.072	0.588	0.100	0.675	2.272	0.103	3.994	2.415	0.000
L1	0.034	0.610	0.107	6.166	2.008	0.000	2.825	2.148	0.000
B	0.075	1.673	0.879	7.806	2.674	0.000	2.191	1.879	0.000
Pog	− 0.259	2.003	0.877	13.228	4.020	0.000	2.003	2.300	0.000
Gn	− 0.250	1.971	0.943	13.213	4.282	0.000	0.306	1.986	0.390
Me	− 0.250	1.971	0.943	12.747	4.123	0.000	− 0.356	1.945	0.308

Since the patients in our study were diagnosed with skeletal Class II malocclusion with a severe retruded mandible and a vertical growth pattern and received LeFort I osteotomy for maxillary impaction, there were no significant changes for maxillary landmarks in the anteroposterior axis. In contrast, the changes were significant for most skeletal mandibular landmarks in the anteroposterior axis (Table 4). There was a significant advancement in lower incisors (ΔL1, 6.166 ± 2.008 mm, *p* < 0.01). The average antedisplacement of anteroposterior correction was 7.806 ± 2.674 mm at B (*p* < 0.01), 13.228 ± 4.020 mm at Pog (*p* < 0.01), 13.213 ± 4.282 mm at Gn (*p* < 0.01), and 12.747 ± 4.123 mm at Me (*p* < 0.01). However, there was no significant setback in the maxillary (point A, *p* > 0.05).

In the vertical axis, there was a significant upward movement both in the maxillary and mandible, and the changes in the maxillary were more evident than the ones in the mandible. In maxillary, the average upward correction was 2.600 ± 3.088 mm, *p* < 0.01 at point A, 3.806 ± 2.491 mm, *p* < 0.01 at ΔU1L and 3.994 ± 2.415 mm, *p* < 0.01 at ΔU1. Additionally, the average upward correction in the mandible was 2.825 ± 2.148 mm, *p* < 0.01 at ΔL1 and 2.191 ± 1.879 mm, *p* < 0.05 at point B. However, none of the landmarks related to the chin showed statistically significant changes (*p* > 0.05).

### Changes in soft tissue landmarks and measurement

Table 5 shows the changes in soft tissue landmarks after orthognathic surgery in facial scan images' transverse, anteroposterior, and vertical axes. In the transverse axis, only the landmarks related to the nose were widened (Al, *p* < 0.01; Ac, *p* < 0.01). There was a significant increase in the width of the nose (ΔWidth of Alare, 2.678 ± 1.671 mm, *p* < 0.01) (Table 6). No significant changes were observed for other points (*p* > 0.05).

**Table 5:** Changes in soft tissue landmarks on 3D stereophotogrammetry images.

Landmarks	ΔT2-T1(transverse)			ΔT2-T1(anteroposterior)			ΔT2-T1(vertical)		
	Mean	SD	*p* value	Mean	SD	*p* value	Mean	SD	*p* value
Ex-r	− 0.500	2.659	0.296	0.572	1.553	0.046	− 0.453	1.233	0.046
Ex-l	0.697	2.603	0.140	0.213	1.000	0.238	− 0.256	0.980	0.149
En-r	0.350	1.186	0.105	0.419	0.991	0.023	0.006	1.081	0.974
En-l	− 0.484	1.064	0.015	0.281	1.335	0.243	0.125	0.691	0.314
N′	0.038	0.212	0.325	0.028	1.128	0.889	0.009	1.325	0.968
Prn	0.141	0.683	0.253	1.125	1.035	0.000	0.703	1.141	0.001
Sn	0.141	0.683	0.253	1.828	1.384	0.000	1.078	1.084	0.000
Al-r	− 1.275	1.134	0.000	2.863	1.608	0.000	1.841	1.273	0.000
Al-l	1.403	0.887	0.000	2.734	1.565	0.000	2.022	2.428	0.000
Ac-r	− 0.044	5.903	0.967	2.619	1.426	0.000	1.281	1.170	0.000
Ac-l	0.975	1.156	0.000	2.866	1.841	0.000	1.441	1.203	0.000
Sbal-r	− 0.106	1.224	0.627	2.069	1.530	0.000	1.356	1.206	0.000
Sbal-l	0.269	1.287	0.246	2.531	1.883	0.000	1.503	1.203	0.000
Ls	0.050	0.653	0.668	1.606	2.490	0.001	1.472	1.946	0.009
Li	0.038	0.647	0.745	4.791	2.232	0.000	3.469	2.732	0.000
Ch-r	0.406	1.239	0.073	2.541	1.788	0.000	1.853	1.780	0.001
Ch-l	− 0.716	1.859	0.037	3.066	1.791	0.000	1.866	1.452	0.000
Sto	0.050	0.438	0.523	2.272	2.532	0.000	1.891	1.736	0.000
Ck-r	− 0.353	2.708	0.466	0.456	1.391	0.073	1.878	1.739	0.001
Ck-l	0.713	2.609	0.132	0.278	1.009	0.129	1.891	1.410	0.000
B′	0.156	0.783	0.268	6.928	2.465	0.000	1.881	3.295	0.003
Pog′	0.044	1.054	0.816	11.534	3.890	0.000	0.213	4.946	0.810
Gn′	0.044	1.054	0.816	13.275	4.848	0.000	− 1.713	4.316	0.032
Me′	0.044	1.054	0.816	14.631	5.610	0.000	− 0.650	3.914	0.355

**Table 6:** Linear changes in soft tissue measurements after orthognathic surgery.

Variables	Mean	SD	p value
ΔWidth of Exocanthion (Ex-r to Ex-l)	1.197	5.179	0.201
ΔWidth of Endocanthion (En-r to En-l)	− 0.834	2.169	0.037
ΔHeight of the nose(N′–Sn)	− 1.069	2.136	0.008
ΔThe length of the bridge of the nose(N-Prn)	− 0.010	1.966	0.978
Δheight of nasal tip(front view)	− 0.375	0.810	0.015
Δheight of nasal tip(lateral view)	− 0.703	0.772	0.000
ΔWidth of Alare (Al-r to Al-l)	2.678	1.671	0.000
ΔWidth of alar curvature (Ac-r to Ac-l)	1.019	6.439	0.378
ΔWidth of nasal Subalare (Sbal-r to Sbal-l)	0.375	2.188	0.340
ΔUpper lip height (Sn–Stms)	− 0.813	1.446	0.340
ΔLip width (Ch dev–Ch ctl)	− 1.122	2.315	0.010
ΔUpper vermilion height (Ls–Stms),	− 0.419	1.580	0.144
ΔLower vermilion height (Li–stmi)	− 1.578	2.653	0.002

In the anteroposterior direction, most nose, lip, and chin landmarks moved forward significantly (*p* < 0.01). The changes in the chin were the most, the lips second, and the nose least. There was a significant decrease in the height of the nasal tip (lateral view) (− 0.703 ± 0.772 mm, *p* < 0.01). The eyes and cheek landmarks exhibited no significant changes (*p* > 0.05).

Additionally, in the vertical axis, there were significant upward movement in the landmarks related to the nose, lips, and cheek (*p* < 0.01) but not in the eyes and chin. And the upward shift of the lower lip was the most, the upper lip second, and the nose the least. There was a significant decrease in upper lip height (− 0.813 ± 1.446 mm, *p* < 0.05) and in lower vermilion height (lower, − 1.578 ± 2.653 mm, *p* < 0.01).

### Correlations and ratio between changes in the hard and soft tissues

Since there was no significant change in the transverse axis, our study did not discuss the correlations and ratios between corresponding hard and soft tissue landmarks.

**Figure 4:**
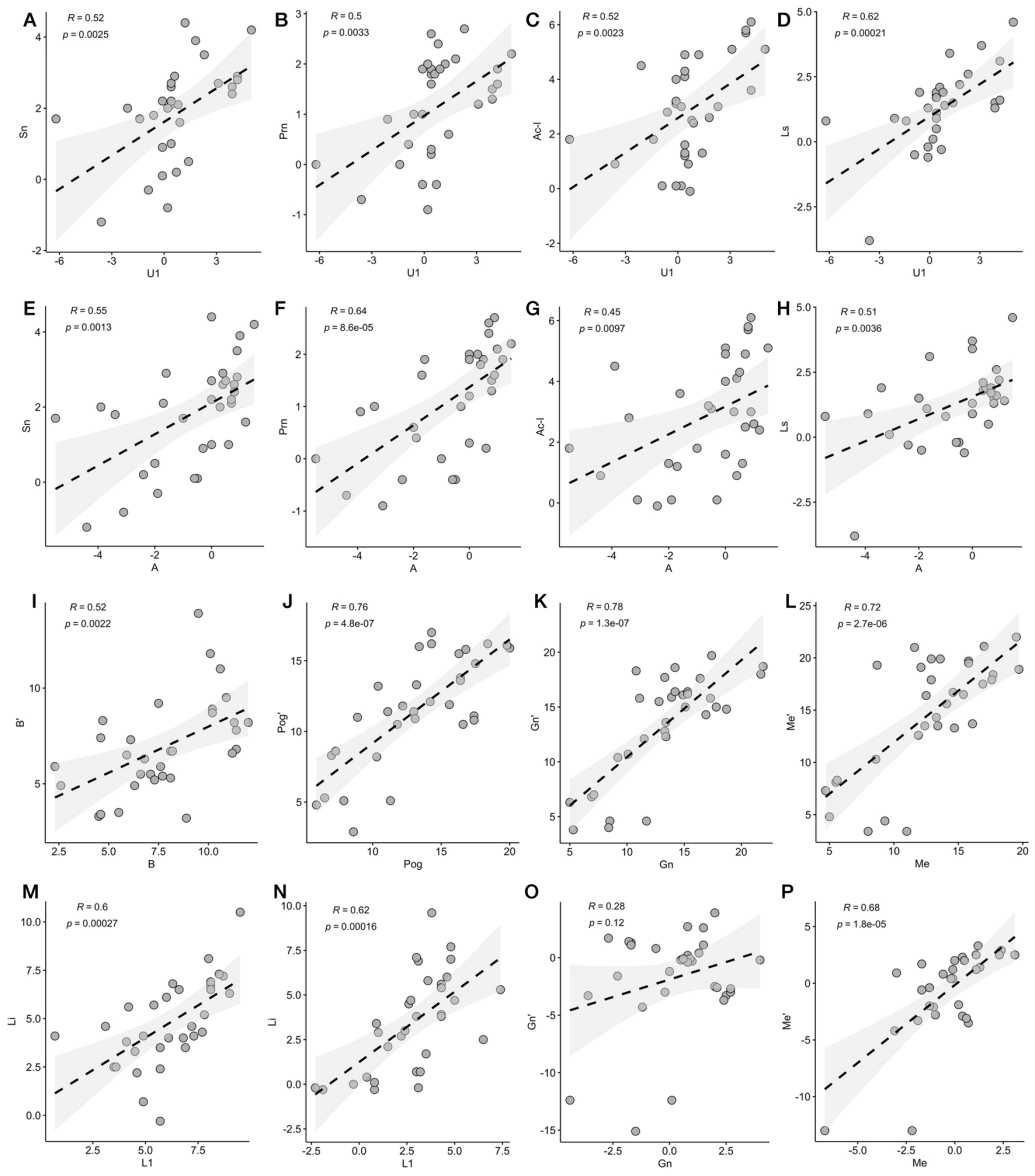
Figures for Pearson correlation. Correlation between soft tissue landmarks (Sn, Prn, Ac-l and Ls) and hard tissue landmarks U1 (**A**–**D**) and A (**E**–**H**) in the anteroposterior axis. Correlation between B′ and B (**I**), Pog′ and Pog(**J**), Gn′ and Gn(**K**), Me′ and Me(**L**), Li and L1(**M**) in the anteroposterior axis. Correlation between Li and L1(**N**), Gn′ and Gn(**O**), Me′ and Me(**P**) in the vertical axis.

In the anteroposterior axis, soft tissue landmarks related to the nose (Sn, Prn, Ac-l) and upper lip (Ls) demonstrated a significant correlation with hard tissue landmarks (A and U1) in the maxilla. And the ratios of maxillary were more than 1(Sn/A 1.431, and Ls/U1 2.930). However, soft tissue landmarks related to the lower lip (Li) in the mandible showed a significant correlation with lower incisors (L1). Correlations between all of the soft tissue and underlying corresponding hard tissue in chin-related landmarks were significant (*p* < 0.01) (Tables 7 and 8, Fig. 4a–m). Furthermore, there was an increasing gradient of ratios from Li/L1 to Me′/Me (Li/L1 0.921, B′/B 0.974, Pog′/Pog 1.103, Gn′/Gn 1.008, Me′/Me 1.169), which meant that the closer the points to the chin, the closer the ratio to 1 (Fig. 4n–p).

**Table 7:** Correlation coefficients (*p*) and ratio of soft-to-hard tissue movement (S/H) in maxillary.

Transverse	A			U1		
	r	*p*	ratio	r	*p*	ratio
Prn	0.006	0.974	0.014	0.203	0.266	0.116
Sn	0.006	0.974	0.014	0.203	0.266	0.166
Ac-r	− 0.046	0.802	− 0.025	− 0.015	0.933	− 3.206
Ac-l	0.305	0.089	0.046	0.243	0.180	0.161
Ls	0.492	0.004	0.055	0.585	0.000	0.024
Anteroposterior	A0.305			U1		
	r	p	ratio	r	p	ratio
Prn	0.638	0.000	1.124	0.504	0.003	0.547
Sn	0.545	0.001	1.431	0.516	0.002	0.588
Ac-r	0.407	0.021	0.942	0.277	0.125	0.127
Ac-l	0.451	0.010	1.757	0.519	0.002	− 0.966
Ls	0.508	0.004	2.323	0.619	0.000	2.930
Vertical	A			U1		
	r	p	ratio	r	p	ratio
Prn	0.290	0.108	0.455	0.017	0.925	0.276
Sn	0.336	0.060	0.305	0.056	0.760	0.462
Ac-r	0.099	0.589	0.423	0.085	0.642	0.425
Ac-l	0.261	0.149	0.463	0.265	0.143	0.449
Ls	0.216	0.235	0.350	0.221	0.224	0.482

**Table 8:** Correlation coefficients (*p*) and ratio of soft-to-hard tissue movement (S/H) in mandible.

Transverse	L1			B			Pog			Gn			Me		
	r	p	ratio	r	p	ratio	r	p	ratio	r	p	ratio	r	p	ratio
Li	0.393	0.026	0.204	0.109	0.552	0.112	0.356	0.046	0.422	0.348	0.051	0.095	0.348	0.051	0.095
B′	0.558	0.001	0.314	0.416	0.018	0.193	0.515	0.003	0.422	0.511	0.003	0.292	0.511	0.003	0.292
Pog′	0.635	0.000	0.414	0.499	0.004	0.263	0.609	0.000	0.422	0.610	0.000	0.424	0.610	0.000	0.424
Gn′	0.635	0.000	0.414	0.499	0.004	0.263	0.609	0.000	− 0.526	0.610	0.000	0.424	0.610	0.000	0.424
Me′	0.635	0.000	0.414	0.499	0.004	0.263	0.609	0.000	0.301	0.610	0.000	0.424	0.610	0.000	0.424
Anteroposterior	L1			B			Pog			Gn			Me		
	r	p	ratio	r	p	ratio	r	p	ratio	r	p	ratio	r	p	ratio
Li	0.602	0.000	0.921	0.595	0.000	0.664	0.678	0.000	0.883	0.645	0.000	0.367	0.655	0.000	0.383
B′	0.437	0.012	1.340	0.522	0.002	0.974	0.667	0.000	0.996	0.586	0.000	0.548	0.591	0.000	0.573
Pog′	0.434	0.013	2.115	0.545	0.001	1.577	0.759	0.000	1.103	0.742	0.000	0.893	0.659	0.000	0.945
Gn′	0.509	0.003	2.358	0.592	0.000	1.792	0.807	0.000	0.215	0.781	0.000	1.008	0.758	0.000	1.056
Me′	0.498	0.004	2.533	0.558	0.001	1.976	0.740	0.000	0.198	0.714	0.000	1.117	0.725	0.000	1.169
Vertical	L1			B			Pog			Gn			Me		
	r	p	ratio	r	p	ratio	r	p	ratio	r	p	ratio	r	p	ratio
Li	0.620	0.000	1.145	0.327	0.068	1.216	0.253	0.163	− 0.544	− 0.012	0.949	− 0.724	0.353	0.047	0.015
B′	0.393	0.026	0.389	0.107	0.561	1.068	0.103	0.574	− 0.053	− 0.062	0.737	− 1.475	0.166	0.362	− 1.469
Pog′	0.387	0.029	− 0.385	0.207	0.254	0.377	0.260	0.151	0.837	0.050	0.786	− 2.522	0.445	0.011	− 0.138
Gn′	0.424	0.016	− 0.795	0.534	0.002	0.127	0.373	0.035	− 0.362	0.280	0.120	0.922	0.532	0.002	0.845
Me′	0.557	0.001	− 0.378	0.526	0.002	− 0.551	0.428	0.015	− 0.325	0.281	0.119	0.722	0.681	0.000	− 0.287

In the vertical axis, there were no significant correlations among maxillary hard tissue landmarks. In the mandible, only the soft tissue and underlying corresponding hard tissue landmarks in the chin showed significant correlations (*p* < 0.01), and the ratios were approximately 1 (Li/L1 1.145, Gn′/Gn 0.922, Me′/Me 0.287) (Table 7 and 8).

## Discussion

This study used CBCT and 3D stereophotogrammetry to study the changes and correlations between hard and soft tissues for skeletal Class II patients after orthognathic surgery. This method has the advantage of three dimensions change measurement by using an identical 3D coordinate system, accurate and precise measurements because of rotating the images in any direction, and high reproducibility. However, there are disadvantages of high cost, the impossibility of acquiring dynamic images, the need to calibrate the system frequently. The accuracy of superimposing the surface image of CBCT and the facial scan has been evaluated in previous studies, which reported that the image fusion was acceptable with a minimum error of less than 1 mm^18,19^. According to the literature, postoperative swelling is resolved by almost 80% after 6 months^20^. Additionally, we analyzed the images taken 6 months after surgery without debonding to minimize any unwanted effects, such as postsurgery swelling and ensure sufficient stabilization of the soft facial tissues.

For hard tissue landmarks, we found significant changes in landmarks only related to the incisors in the transverse axes. These changes might be due to no significant asymmetry observed in our patients. In anteroposterior axes, there was a significant advancement for the landmarks of the mandibular and chin, as reported in other articles about surgical treatment of skeletal Class II patients, who had their mandibular and chin positioned anteriorly after mandibular advancement surgery^4,21^. However, we did not noticed a significant significant setback in the maxillary, and this might be due to the selection criteria included in this study. In our study, most patients were diagnosed with mandible hypoplasia with a severe retruded mandible and a vertical growth pattern, one piece Le Fort I osteotomy was conducted mainly for maxillary impaction. There was a counter clock rotation and an upward shift of the forepart of the maxillary other than setback movement for these patients. In our study, many patients had the advancement of point A in the anteroposterior axis. Notably, this was correlated with the upward movement of the landmarks of the incisors (U1L, U1, and L1) and point A and point B in the vertical axes. However, no landmarks related to the chin showed statistically significant changes. This might be because of the hard-tissue vertical reduction at the chin after genioplasty, which was consistent with the results of Sylvain Chamberland, who reported that vertical soft-tissue change of chin points was less predictable after isolated functional genioplasty^5^.

The changes in soft tissue landmarks were correlated with the changes in inferior hard tissue. There was a significant forward and upward movement of the landmarks of the nose and lip. Notably, these might be due to the counter clock rotation and upward shift of the forepart other than the setback movement of the maxillary. The significant decrease in nasal tip height (lateral view) meant that Sn's forward movement was more than that of Prn. Likewise, the decrease in upper lip height meant a lesser upward shift of Sn than Ls. This may be because the Sn point was closer to the osteotomy line, and the soft tissues underlying these points were thin. A significant decrease in upper and lower vermilion height could be explained by a decrease in soft tissue tension after orthognathic surgery because stretching of the lip area's soft tissue can be reduced as the changes after retraction^22^. Our results showed a significant increase in nose width, usually noticed after maxillary advancement surgery^14,23^ but seldom reported in previous studies about skeletal Class II patients. This finding suggested that the counter clock rotation and upward shift of the forepart, other than the setback movement of the maxillary, would push forward the nasal base, contributing to the uplift of the soft tissue around the nose and upper lip, leading to the broadening of nasal width. Another potential factor contributing to the widening of the alar bases was soft tissue dissection. Periosteal elevation will sever necessary muscular attachments leading to muscular retraction, alar flaring and shortening, and flattening and thinning of the upper lip^24^. In some cases, a combined alar cinch suture was recommended to control alar flaring at the time of surgery^25^.

For the correlations between changes in the hard and soft tissues, a significant correlation was observed between soft-tissue and hard-tissue changes only in the anteroposterior axis for maxillary. The ratios of maxillary were more than 1(Sn/A 1.431, Ls/U1 2.930) in our study. However, the respective measurements in the study by Suyun Seon and Hyun-Woo Lee were 0.298(ΔSN/ΔA)^26^ and − 0.23 in the study conducted by Zhou Zhijie^27^. The big difference might be due to a counter clock rotation and an upward shift of the forepart of the maxillary other than setback movement for half of the patients in this study. The mean maxillary advancement and impaction amounts were − 0.675 and 2.600 mm in this study, respectively, while they were 1.84–1.54 mm in the study by Zhou Zhijie^27^. The upward shift of the maxillary in this study caused the forward movement of Sn and Ls. Furthermore, in the vertical axis, the ratio of Prn and point A (0.455) was consistent with the results of Suyun Seon (0.647)^26^, although there was no significant relationship between them.

For the mandible, there was an increasing gradient of ratios from Li/L1 to Me′/Me in the anteroposterior axis, which was consistent with the studies of A.S. Storms, who found that the lower lip followed the lower incisor to a lesser extent than Pog′:Pog, B′:B, and Me′:Me (which approached a 1:1 relationship)^26^. These findings suggest that the lower lip could be under the influence of the muscle rather than the bone^29,30^, and this might be related to the inherent differences in the soft tissue between the lip and chin. Additionally, the ratios of the chin were also consistent with the results of Alexander Bral, who found an sPg:Pg ratio of 87% for movement in the anterior direction among the patients who underwent BSSO and a higher ratio of 102% among patients who underwent bimaxillary surgery^4^. A systematic review also had ratios ranging from 71 to 110% of included studies, and the average ratio was 100% when BSSO was performed to produce anterior displacement^31^. The small differences could be due to differences in patient populations or errors in measurement. In the vertical axis, the ratios were approximately 1. However, a systematic review studying the hard and soft tissue response following isolated genioplasty reported that the ratio of soft and hard tissue changes after genioplasty ranged from 0.09 to 0.7 among the studies in the vertical plane^32^. Furthermore, Sylvain Chamberland revealed that chin soft-tissue changes could be predicted horizontally more precisely than vertically^5^. Therefore, more studies were needed to analyze the relationship between the soft and hard tissues of the chin in the vertical axis.

This study showed the 3D soft and hard tissue changes after bimaxillary surgery in Class II patients using CBCT and facial scanning. The results of this study would contribute clinicians to predict the effect of orthognathic surgery for soft tissue. It could guide surgical planning to improve patient outcomes. As machine learning and artificial intelligence participated more in the guidance of therapeutic approaches^33–35^, We hoped the results of this study would contribute to the database of hard and soft tissue changes in skeletal class II patients with high mandibular plane angle undergoing orthognathic surgery, which could further facilitate the precise prediction of the effect of orthognathic surgery for soft tissue.

However, there are some limitations in this retrospective study. A double-blinded, randomized controlled trial is the ideal experimental design for a comparative study. However, because orthognathic surgery is performed according to the patients' profile and occlusion, randomization of the indications for orthognathic surgery is impossible. Another limitation was the small sample size. A larger sample size would be needed in future studies. What’s more, we could only evaluate short-term changes 6 months after surgery in our study, a long-time evaluation should be performed to study skeletal relapse after orthognathic surgery. Notably, Mobarak et al.^36^ reported 33% of relapse at Pog 3 years. A systematic review^37^ also reported skeletal relapse in the long-term, with 2–31.4% after 1 year and 60% after 12.7 years. Consequently, skeletal relapse is a complex multifactorial process. Many factors, such as amount of advancement, mandibular plane angle, distal segment rotation, seating of the condyles, type of fixation, soft tissue and muscle stretch, and surgeon skills, may influence skeletal relapse. Furthermore, more precise treatment planning could be done if the studies could consider it.

## Conclusions

We found that the 3D soft tissue profile was noticeably improved following bimaxillary surgery for Class II patients. In addition, CBCT and 3D stereophotogrammetry offered a convenient and alternative way to evaluate postsurgical soft tissue changes. The 3D analysis enabled us to understand hard and soft tissues quantitatively, providing helpful information for orthognathic treatment planning for skeletal class II patients. Our further research should focus on increasing the sample size to establish a more powerful facial prediction-system for Class II patients following bimaxillary surgery.

### Supplementary Information

Supplementary Information 1.
